# Therapeutic targeting of *TP53* nonsense mutations in cancer

**DOI:** 10.48101/ujms.v129.10719

**Published:** 2024-05-27

**Authors:** Charlotte Strandgren, Klas G. Wiman

**Affiliations:** Karolinska Institutet, Departement of Oncology-Pathology, Stockholm, Sweden

**Keywords:** *TP53*, nonsense mutations, translational readthrough, cancer therapy

## Abstract

Mutations in the *TP53* tumor suppressor gene occur with high prevalence in a wide range of human tumors. A significant fraction of these mutations (around 10%) are nonsense mutations, creating a premature termination codon (PTC) that leads to the expression of truncated inactive p53 protein. Induction of translational readthrough across a PTC in nonsense mutant *TP53* allows the production of full-length protein and potentially restoration of normal p53 function. Aminoglycoside antibiotics and a number of novel compounds have been shown to induce full-length p53 in tumor cells carrying various *TP53* nonsense mutations. Full-length p53 protein generated by translational readthrough retains the capacity to transactivate p53 target genes and trigger tumor cell death. These findings raise hopes for efficient therapy of *TP53* nonsense mutant tumors in the future.

## Nonsense mutations in human diseases

Nonsense mutations have a causative role in various human genetic diseases (1, 2). In cystic fibrosis, nonsense mutations in the cystic fibrosis transmembrane conductance regulator (*CFTR*) gene occur in 10–15% of the cases, leading to disturbances in the respiratory, digestive and reproductive systems (3). Similarly, nonsense mutations that inactivate the dystrophin gene occur in Duchenne muscular dystrophy (4), spinal muscle atrophy (5), and various other inherited diseases (1). Nonsense mutations are also frequent in cancer. While oncogenes that drive cancer growth, for example, *KRAS* and *IDH1*, most often carry activating and recurrent missense mutations, tumor suppressor genes that normally protect from cancer show a high frequency of inactivating nonsense and frame-shift mutations (1, 6). The *TP53* tumor suppressor gene is mutated in around 50% of all human tumors and around 10% of these mutations are nonsense mutations (the COSMIC database) (7).

**Figure 1:**
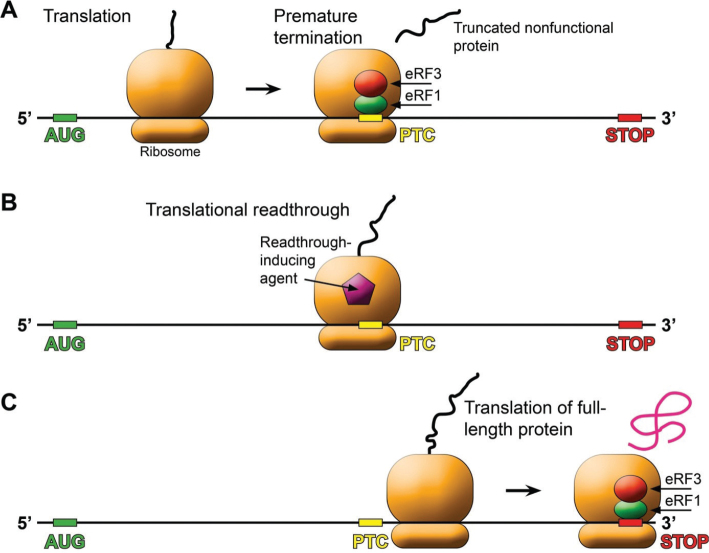
Overview of translational readthrough. (a) Translation is stalled when the ribosome reaches a premature termination codon (PTC) in the mRNA. Release factors eRF1 and eRF3 bind and translation is prematurely terminated, causing the release of a truncated non-functional protein. (b) Aminoglycoside antibiotics and other readthrough-inducing agents can suppress the PTC by enabling misincorporation of a near-cognate aminoacyl-tRNA. (c) Readthrough induction allows the ribosome to continue translation across the PTC until the normal stop codon, resulting in the production of a full-length protein.

A nonsense mutation gives rise to a premature termination codon (PTC) in the mRNA. When the ribosome encounters a PTC, translation is stalled and release factors eRF1 and eRF3 are recruited, resulting in premature termination of translation and production of a truncated inactive protein (Fig. 1A). mRNAs that carry nonsense mutations are subjected to nonsense-mediated decay (NMD) (8).

## Translational readthrough

Aminoglycoside antibiotics can cause mistranslation of mRNA and suppress nonsense mutations in bacteria and yeast (9, 10), and have also been shown to suppress nonsense mutations in mammalian cells (11). They bind to the decoding center of the ribosome and interfere with codon-anticodon base pairing during translation, allowing insertion of a near-cognate aminoacyl-tRNA at the position of the PTC, leading to continued translation in the proper reading frame and synthesis of a full-length protein (8). This process is referred to as stop codon readthrough or translational readthrough (Fig. 1B,C). These findings have raised hopes for efficient therapy of diseases caused by nonsense mutations. However, aminoglycosides have severe side effects, mainly nephrotoxicity and ototoxicity, that make them unsuitable for long-term therapy. Therefore, attempts have been made to identify novel readthrough-inducing agents with lower toxicity.

The compound PTC124 (Ataluren) was identified in a high-throughput screen using cells expressing a nonsense-mutant firefly luciferase reporter (12). It has shown activity in a mouse model with a human *CFTR*-G542X nonsense mutant allele (13). However, PTC124 was shown to bind and stabilize luciferase, suggesting that the observed activity could be due to stabilization of full-length luciferase that arises from basal readthrough (2). PTC124, under the name Translarna, was granted orphan designation by the EMA in 2005 for treatment of patients with Duchenne muscular dystrophy. However, due to failure in confirming the effectiveness of the medicine in this patient group, the EMA has recently recommended non-renewal of authorization in the EU of Translarna (ema.europa.eu).

An alternative approach for readthrough induction is targeting of release factors eRF1 and eRF3. Screening of more than 770 000 compounds using a nonsense mutant NanoLuc reporter in fusion with the human beta-globin gene containing two introns with sequences that induce exon junction complex-mediated NMD yielded the compound SRI-37240 (14). The analog SRI-41315 was shown to have even higher potency. Both compounds suppressed PTCs in the *CFTR* gene, and restored expression and function of CFTR. This effect was exerted by reducing the levels of the eRF1 protein via proteasomal degradation. Combination treatment with aminoglycoside G418 resulted in a synergistic increase in CFTR activity as assessed by CFTR-mediated conductance.

Suppressor tRNAs (sup-tRNAs) represent an entirely different strategy. It is based on modifying natural tRNAs by altering the anticodon to decode a specific stop codon, and modulating sequences outside the anticodon to achieve high readthrough efficiency. A recent study demonstrated that such modified sup-tRNAs allow potent translational readthrough of nonsense mutations in the *CFTR* gene and restoration of CFTR expression *and function in nasal epithelial cells from cystic fibrosis patients* (15).

## Compounds that promote readthrough of nonsense mutant *TP53*

**Figure 2:**
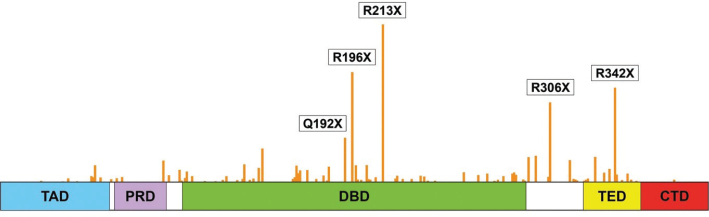
*TP53* nonsense mutation spectrum in cancer. Bars indicate nonsense mutations in the *TP53* coding sequence. Around 10% of all *TP53* mutations are nonsense mutations. The five most common nonsense mutations are indicated. *TP53* nonsense mutations are distributed over most of the p53 coding sequence, in contrast to missense mutations that cluster in the DNA-binding core domain (not shown). The p53 protein (393 amino acid residues) is shown schematically with structural domains: N-terminal transactivation domains (TAD, blue), proline-rich domain (PRD, purple), DNA-binding domain (DBD, green), tetramerization domain (TED, yellow), and C-terminal oligomerization domain (CTD, red). Data were collected from the COSMIC database (6 April 2024, cancer.sanger.ac.uk).

As indicated above, a significant fraction of *TP53* mutations in cancer are nonsense mutations. According to WHO statistics (who.int/health-topics/cancer), close to 1 million new cases of cancer diagnosed each year are estimated to carry nonsense mutant *TP53*. The most common *TP53* nonsense mutation is R213X (Figure 2), which is among the 10 most frequent *TP53* mutations in cancer.

Can translational readthrough be applied for the treatment of tumors that carry nonsense mutant *TP53*? This possibility has been addressed in several studies. Keeling and Bedwell first demonstrated translational readthrough of mouse *Trp53* nonsense mutations including *Trp53*-R210X (corresponding to human *TP53*-R213X) using different aminoglycoside antibiotics, confirmed production of full-length active p53 protein, and found that amikacin was the most potent readthrough inducer (16). Bidou and colleagues examined induction of translational readthrough of human nonsense mutant *TP53* and showed expression of full-length p53 protein upon treatment with aminoglycoside antibiotics G418 and Gentamicin (17). This study assessed readthrough of 11 *TP53* nonsense mutations (including the most common mutations R213X, R196X and R306X), and among these, R213X showed the highest readthrough efficiency. Aminoglycoside-induced translational readthrough of the R213X and Q192X mutants generated active p53 protein capable of inhibiting cell growth and triggering cell death by apoptosis.

The aminoglycoside antibiotic gentamicin is known to contain a mixture of several aminoglycosides. Examination of their readthrough efficacy on R213X nonsense mutant *TP53* in HDQ-P1 human breast carcinoma cells identified one minor component, gentamicin X2, as the most active component (18). Gentamicin X2 also showed less severe nephrotoxicity than G418 *in vivo* upon administration in rats. This suggests that gentamicin X2 should be explored further for therapeutic readthrough induction.

ELX-02 is a synthetic eukaryotic ribosome-selective glycoside previously named NB124 (19). Studies in DMS-114 human small-cell lung cancer cells carrying R213X nonsense mutant *TP53* demonstrated substantial induction of full-length p53 protein levels. Proteomic analysis supported the conclusion that ELX-02 has little effect on natural stop codons in neither DMS-114 cells nor white blood cells from healthy individuals (20). A phase II trial with ELX-02 in cystic fibrosis patients with G542X nonsense mutant *CFTR* gene has been completed but no results have been posted (ClinicalTrials.gov; NCT04135495). A phase II trial in patients with nephropathic cystinosis with nonsense mutant *CTNS* gene has been terminated (ClinicalTrials.gov; NCT04069260).

The adenosine nucleotide analog clitocine was identified in a high-throughput screen using a nonsense mutant luciferase reporter (21). Clitocine is incorporated into mRNA in place of adenosine and was shown to promote translational readthrough if present at the third position of a PTC. Moreover, clitocine induced readthrough of Q136X, R196X and W146X nonsense mutant *TP53* and expression of full-length p53 protein in human tumor cell lines, followed by upregulation of the p53 target gene *CDKN1A* (p21) and activation of caspase 3 and 7, indicating that full-length p53 induced by clitocine is transcriptionally active. Finally, clitocine inhibited the growth of CAOV-3 human ovarian carcinoma cell xenografts carrying Q136X nonsense mutant *TP53* in mice upon subcutaneous administration of the compound (21).

The purine derivative 2,6-diaminopurine (DAP) was identified as the active component of an extract of the mushroom *Lepista inversa*, using a firefly reporter gene with a nonsense mutation and a downstream intron, making the mRNA a target for NMD. DAP showed higher readthrough efficiency than G418 but was active only on UGA PTCs. *In vivo* studies in mice showed that DAP inhibits the growth of Calu-6 human tumor xenografts carrying R213X nonsense mutant *TP53*. Readthrough induced by DAP resulted in the exclusive insertion of a tryptophan residue at the PTC. Interestingly, DAP was shown to inhibit the methyltransferase FTSJ1 that methylates a specific cytosine in Trp tRNA, thereby allowing its increased miscoding of UGA PTCs (22).

The eukaryotic release factors eRF1 and eRF3 facilitate termination of translation (8). eRF1 binds to any of the three termination codons in the ribosomal A site and interacts with eRF3, leading to release of the polypeptide from the ribosome. Depletion of eRF1 or eRF3 by siRNA knockdown or antisense oligonucleotides can stimulate translational readthrough (23). CC-885 and CC-90009 are small molecules that target eRF3 for proteosomal degradation via binding to cereblon (24, 25). Both compounds had a modest readthrough activity on R213X nonsense mutant *TP53* (UGA PTC) in HDQ-P1 cells, but combination with G418 resulted in pronounced synergistic induction of full-length p53. Similarly, combination treatment showed synergy in SW900 cells carrying Q167X nonsense mutant *TP53* (UAG PTC), but much lower readthrough activity in Caov-3 cells carrying the *TP53*-Q136X nonsense mutation (UAA PTC) (26).

Bidou and colleagues performed high-throughput screening of chemical libraries of >17,000 compounds based on NIH3T3 cells transfected with a luciferase reporter gene carrying the *TP53* nonsense mutation R213X (27). This screen led to the identification of the compound 2-guanidino-quinazoline (TLN468, named translectin) which induced full-length p53 levels in human *TP53*-R213X nonsense mutant HDQ-P1 cells in a dose-dependent manner. The full-length protein was at least partially active as transcription factor, as demonstrated by activation of a luciferase reporter gene driven by a p53-dependent promoter and upregulation of endogenous Bax, a p53 target gene, in HDQ-P1 cells. This study further showed that TLN468 has readthrough activity on common nonsense mutations in the Duchenne muscular dystrophy gene (*DMD*) but no major readthrough effect on normal termination codons, as assessed by Ribo-seq.

We have performed an *in silico* analysis of data available at the NCI database to search for compounds or approved drugs with potential readthrough activity, using a simple algorithm to identify agents with preferential effect on tumor cell lines carrying nonsense mutant *TP53* (28). This led to the identification of the well-known chemotherapeutic drug 5-fluorouracil (5-FU) as a readthrough inducer and the 5-FU metabolite 5-fluorouridine (5-FUr) as the active metabolite. Full-length p53 induced by 5-FUr can upregulate p53 target genes including *CDKN1A, PUMA* and *ZMAT3*, and induce p53-dependent cell death. Ribo-seq analysis supported readthrough at the R213X PTC. Like clitocine, 5-FUr is incorporated into mRNA, suggesting a similar mechanism of action. Indeed, replacement of uracil (U) in mRNA by 5-FUr may allow base-pairing with C (29), which would change a UGA PTC to the Arg codon CGA and thus promote readthrough by insertion of an Arg residue. We proposed a dual mechanism of action for 5-FUr: 1) Induction of readthrough by incorporation of 5-FUr into the UGA PTC, and 2) Further stabilization of full-length p53 by cellular stress induced by 5-FUr (28).

We also performed high-throughput screening of chemical libraries to identify novel readthrough inducers. Our screen was based on p53 immunofluorescence staining of HDQ-P1 cells homozygous for the *TP53*-R213X nonsense mutation. Two compounds, C47 and C61, were selected for further characterization. While both compounds had a relatively moderate effect on R213X nonsense mutant *TP53* by themselves, C47 showed significant synergy with G418 for induction of full-length p53, and C61 was synergistic in combination with eRF3 degraders CC-885 and CC-90009. Additionally, C47 induced full-length PTEN protein in human glioblastoma cells carrying endogenous nonsense mutant *PTEN* (30) (see below).

## Readthrough of nonsense mutations in other cancer-associated genes

Translational readthrough has been explored as a strategy for targeting several other nonsense mutant tumor suppressor genes. The APC tumor suppressor protein inhibits cell growth through negative regulation of beta-catenin activity (31). Mutations in the *APC* gene occur in more than 80% of colorectal tumors, and around 30% of these mutations are nonsense mutations (32, 33). Aminoglycosides Geneticin (G418) and Gentamicin and the macrolide antibiotic tylosin were shown to induce translational readthrough of an *APC* nonsense mutation (R1450X) in a reporter construct. These antibiotics also induced levels of full-length APC protein and inhibited growth and survival of SW1417 colon carcinoma cells carrying R1450X nonsense mutant *APC*, but had little effect on HCT116 colorectal carcinoma cells carrying wild-type *APC*. Anti-tumor effects were also observed *in vivo* in mice. G418, Gentamicin and Tylosin all inhibited the growth of HT29 tumor cells carrying nonsense mutant *APC* in xenografts in athymic mice, and tylosin inhibited intestinal polyp number and size and prolonged the lifespan in *Apc^Min^* mice that carry L850X nonsense mutant *Apc* (34).

Another tumor suppressor gene that frequently carries nonsense mutations in cancer is *PTEN*. This gene encodes a lipid phosphatase that dephosphorylates phosphatidylinositol (3,4,5)‐triphosphate (PIP3) and thereby antagonizes the PI3 kinase/Akt kinase pathway that drives cell growth (35). The most common *PTEN* nonsense mutations in both somatic and germline tumors are R130X, R233X and R335X. Aminoglycoside antibiotics were shown to induce readthrough of all three *PTEN* nonsense mutants as assessed by expression of full-length PTEN protein in transfected CCOS-7 or U87 cells, with G418 as the most efficient readthrough inducer. Functional assays based on the assessment of Akt phosphorylation and nuclear localization confirmed that translational readthrough generated fully active PTEN protein (36). Studies in our lab confirmed the translational readthrough effect of G418 on these three frequent *PTEN* nonsense mutations, and also demonstrated a synergistic readthrough activity of G418 in combination with C47, a novel molecule identified in a chemical library screen (30).

Rb1, the retinoblastoma tumor suppressor gene product, regulates G1 phase cell cycle progression by controlling the E2F family of transcription factors (37). A major fraction (approx. 25%) of tumor-associated somatic mutations in the *RB1* gene are nonsense mutations, with R251X, R579X and R320X being the most frequent (the COSMIC database) (7). We examined the ability of G418 to induce full-length Rb1 protein in human tumor cells carrying R251X, R320X, R579X or Q702X nonsense mutant *RB1* and observed variable levels of induction; UGA PTCs (R251X, R320X, R579X) showed more robust readthrough than UAA (Q702X). Moreover, the readthrough effect of G418 on R579X nonsense mutant *RB1* was enhanced by combination with the eRF3 degrader CC-90009 (38).

## Challenges with translational readthrough of nonsense mutant *TP53*

Induction of translational readthrough as a therapeutic strategy for *TP53* nonsense mutant tumors faces a number of challenges. First, while several compounds have been shown to induce readthrough and expression of a full-length p53 protein, the efficiency of this process, as evident from the ratio between truncated and full-length protein, varies and may not be sufficient for full functional restoration and induction of cancer cell death *in vivo*. Combination treatment that enhances levels of full-length p53 induced by translational readthrough, as exemplified by the combination of aminoglycosides with the proteasome inhibitor bortezomib or the Mdm2 inhibitor Nutlin 3a (39), could be a fruitful strategy to overcome this obstacle. Second, it is not exactly clear which amino acid is inserted at the PTC upon readthrough. This is relevant to the question whether full-length p53 produced upon readthrough is fully functional. Several of the compounds shown to induce full-length p53 in cancer cells with the *TP53* R213X nonsense mutation, for example, G418, DAP and 5-fluorouridine, were also shown to induce transcriptionally active p53 (17, 21, 22, 28), suggesting that either a large fraction of the full-length p53 carries a wild-type residue at the position of the PTC (in the case of *TP53* R213X, Arg) or that inserted non-cognate residues are compatible with wild-type p53 function. It is worth noting that missense mutations at codon 213 in *TP53* are relatively infrequent (the COSMIC database) (7), consistent with the possibility that amino acid substitutions at this position do not necessarily inactivate p53. Novel engineered tRNAs (sup-tRNAs) may allow both high readthrough efficiency and incorporation of a specific desired amino acid (15). Another important question is to what extent normal termination codons are affected by readthrough. Since normal termination codons show more robust termination of translation due to their proximity to the poly A-tail and poly (A)-binding protein (PABP) that interacts with eRF3 during termination of translation (2), it is conceivable that a therapeutic window exists that allows sufficient levels of readthrough at the disease-causing PTC without any major impact on normal translation termination. This notion is supported by Ribo-seq and proteomic analyses (20, 27, 28).

## Conclusions

Induction of translational readthrough has emerged as a promising strategy for the treatment of diseases caused by nonsense mutations, such as cystic fibrosis, Duchenne muscular dystrophy, and cancer. A number of compounds have been shown to promote translational readthrough across PTCs by various mechanisms and induce expression of full-length protein. In the case of nonsense mutations in the tumor suppressor gene *TP53*, several compounds have demonstrated readthrough activity and induction of full-length p53 that retains the capacity to upregulate p53 target genes and trigger tumor cell death. Further studies are needed to assess readthrough and anti-tumor efficacy of such compounds *in vivo* in suitable animal models. Nevertheless, these findings raise hopes for novel and more effective therapy for a large number of cancer patients with nonsense mutant *TP53*-carrying tumors in the future.
